# Supplementary figures and images for: T cell stimulation remodels the latently HIV-1 infected cell population by differential activation of proviral chromatin
Source: PLoS Pathog. 2022 Jun 6;18(6):e1010555. doi: 10.1371/journal.ppat.1010555 (PMC9203004; doi:10.1371/journal.ppat.1010555)

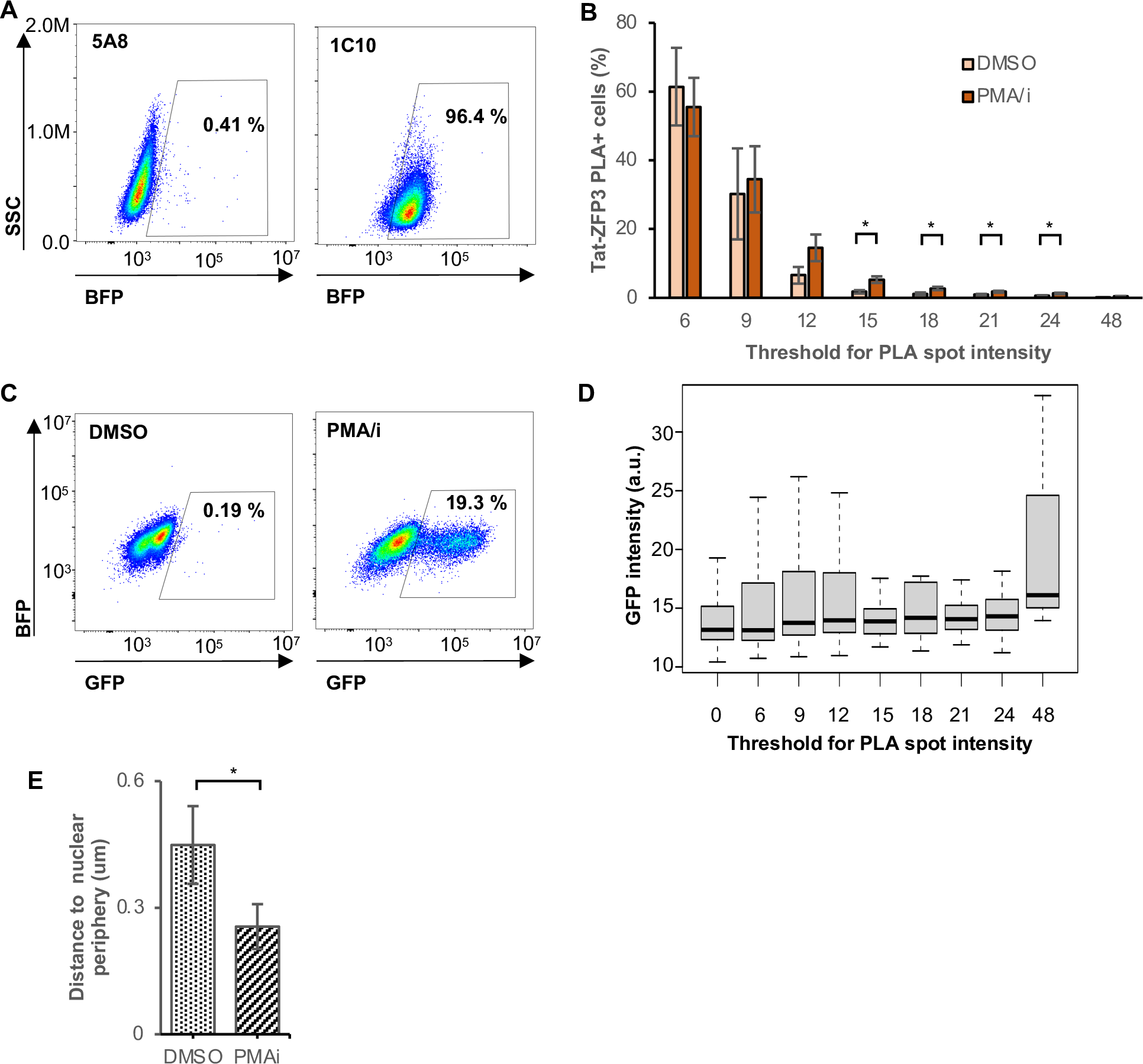

Supplement: S1 Fig — (A) Flow cytometry of 5A8 and 1C10 cells, showing BFP (V450-A) against SSC (SSC-A). (B) Boxplot with the GFP levels relative to intensity of the PLA spot. (C) GFP intensity in Tat-ZFP3 PLA+ cells after 16 h treatment with DMSO or PMA/i. Dotted line shows the background cellular GFP intensity. (D) Flow cytometry of 1C10 cells showing GFP (B525-A) against BFP (V450-A) in unstimulated (DMSO) or stimulated (PMA/i) cells after 24 h (E) Distance between the PLA spot and the nuclear periphery, n = 4 (F) Genome browser view of the MAT2A locus, harboring the HIV-1 provirus. (TIF) [file ppat.1010555.s003.tif]

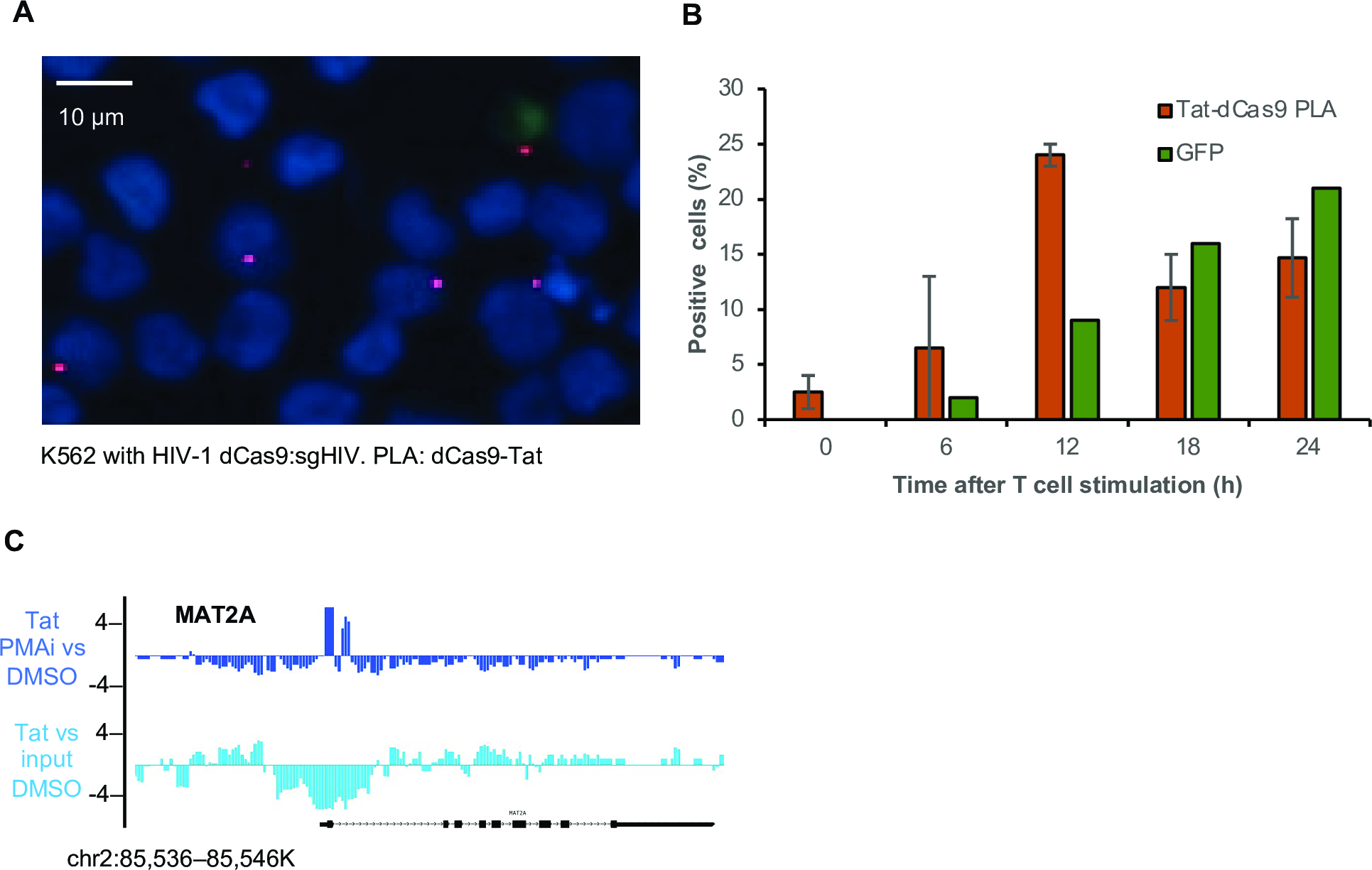

Supplement: S2 Fig — (A) Micrograph of PLA using anti-Tat and anti-HA in a K562 cell line with dCas9-HA and latent HIV-GFP. Cells were also transfected with a plasmid to express a sgRNA targeting the 5´ region of the HIV provirus. (B) Quantification of Tat-dCas9 PLA+ cells and GFP+ cells in time after T cell stimulation by antibodies against CD3 and CD28. (TIF) [file ppat.1010555.s004.tif]

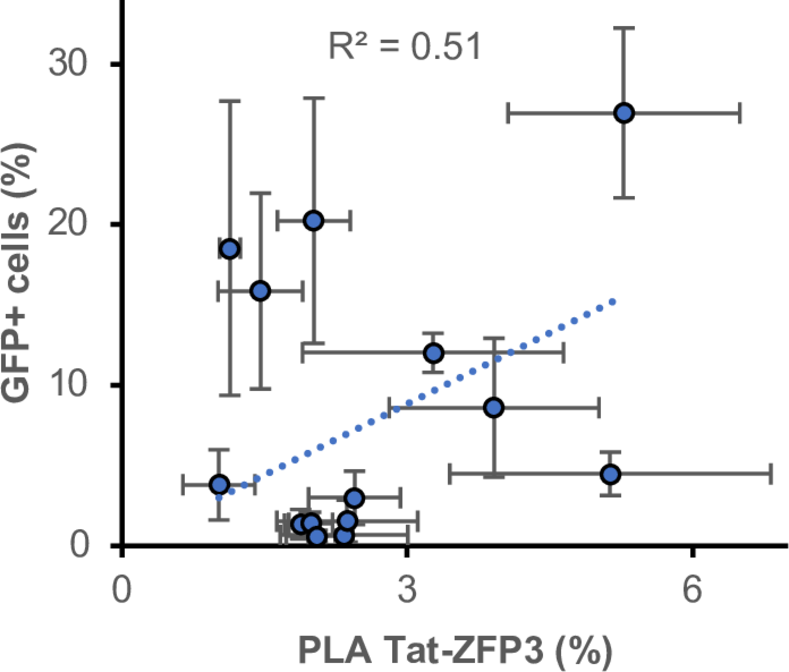

Supplement: S3 Fig — (A–B) Response to latency reversal agents (LRAs) in J-lat 1C10 detected by PLA Tat-ZFP3 (A) and GFP (B). (C) Correlation between GFP and PLA spot. n = 5, error bars show s.e.m.. (TIF) [file ppat.1010555.s005.tif]

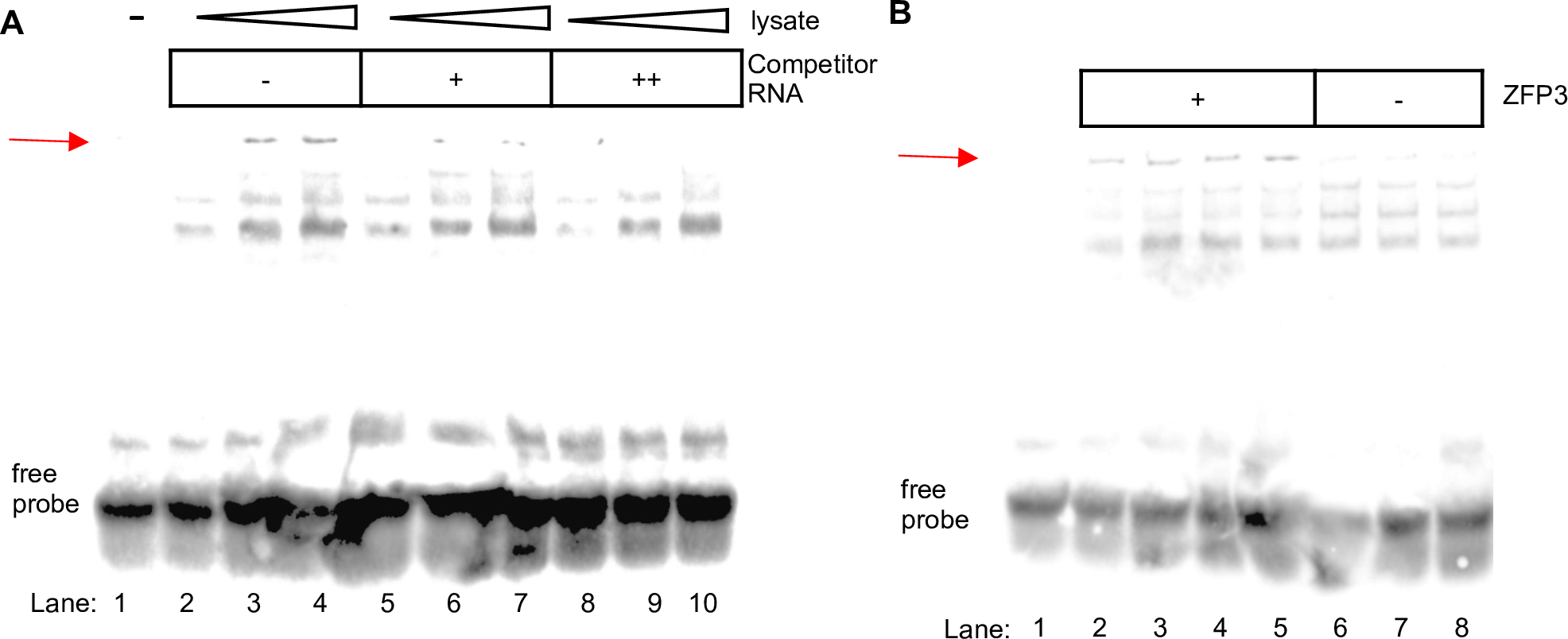

Supplement: S4 Fig — RNA EMSA using a biotinylated HIV LTR RNA probe. Red arrows point to the specific band that appears gel shifted by ZFP3. (A) Gel with no lysate (lane 1) and increasing lysate concentration (lanes 2–4, 5–7, 8–10), and including no competitor (-, lanes 2–4), low (+, lanes 5–7) and high (++, lanes 8–10) concentration of non-biotinylated competitor HIV LTR RNA. (B) Gel with no lysate (lane 1), cell lysates from 293T cells transfected with ZFP3+ (lanes 2–5) and parental ZFP3- (lanes 6–8) plasmid. (TIF) [file ppat.1010555.s006.tif]

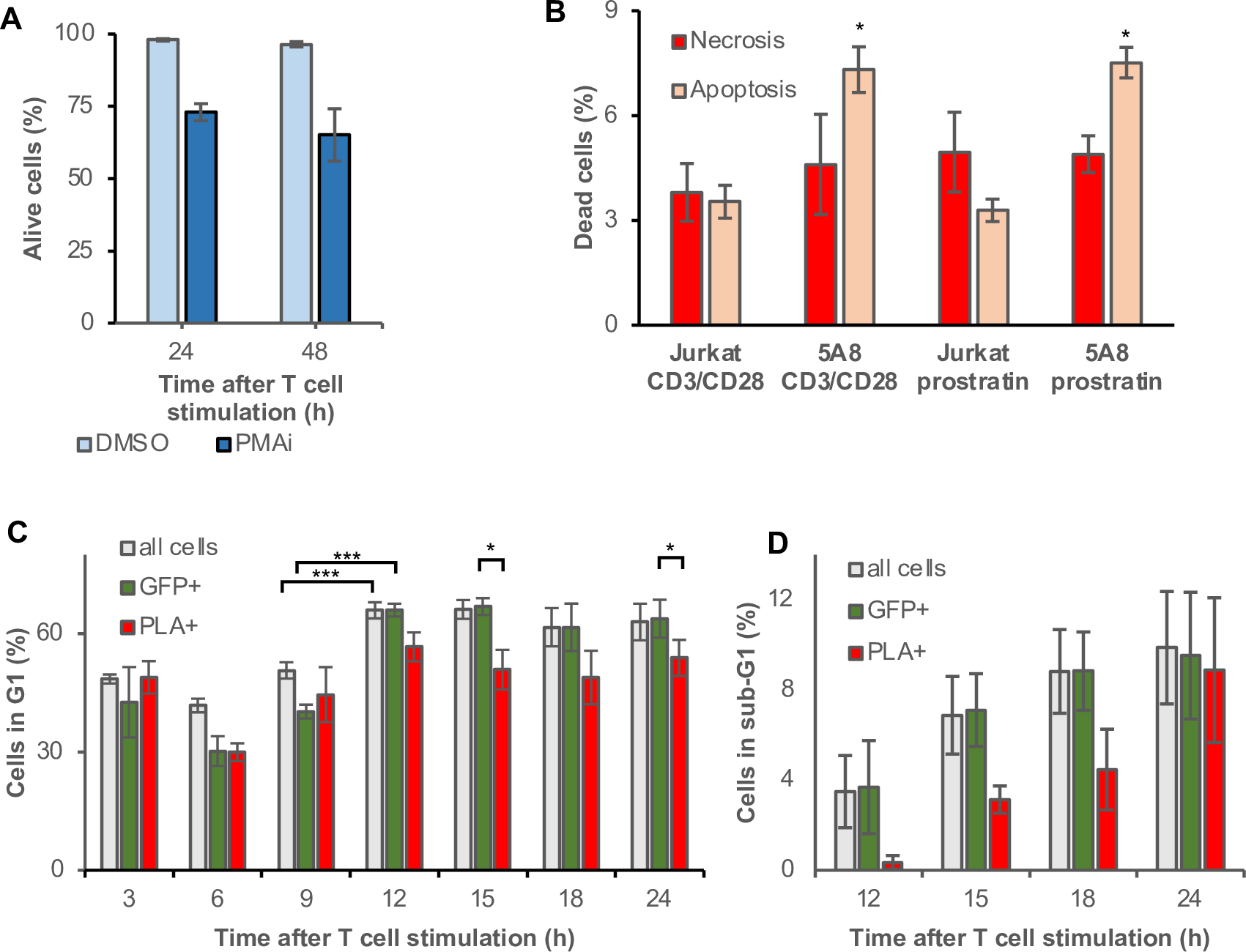

Supplement: S5 Fig — (A) Percentage of live 5A8 cells after T cell stimulation recorded by live/dead cell stain using flow cytometry (n = 3). (B) Apoptosic and necrotic cell populations determined by PI/Annexin V staining and flow cytometry in J-lat 5A8 cells and parental Jurkat cells after 16 h T cell stimulation with CD3-CD28 antibodies or prostratin. * p<0.05 compared to DMSO-treated control. (C) Percentage of 1C10 cells in G1 phase of the cell cycle. All cells in white, Tat-ZFP3 PLA+ cells in red, GFP+ cells in green, n = 5 error bars represent s.e.m., Student t-test p-values * p<0.05. (TIF) [file ppat.1010555.s007.tif]
