# Supplementary material for: T cell stimulation remodels the latently HIV-1 infected cell population by differential activation of proviral chromatin
Source: PLoS Pathog. 2022 Jun 6;18(6):e1010555. doi: 10.1371/journal.ppat.1010555 (PMC9203004; doi:10.1371/journal.ppat.1010555)
Supplement: Attachment — Submitted filename: 121052_1_rebuttal_2273911_r6273f.pdf [file ppat.1010555.s008.pdf]

Full Revision

Review COMMONS

Manuscript number: RC-2021-01086
Corresponding author(s): Peter, Svensson

1. General Statements

This section is optional. Insert here any general statements you wish to make about the goal of the study or about the reviews.

In the revised manuscript we have addressed all of the reviewers' concerns. The conclusions and significance of the study have been strengthened by new experiments, new analysis as well as new connections to previous studies. The only suggested experiment we have not performed is the humanized mouse model suggested by reviewer 3, which we deem is out of scope for the revision. We now stress the general nature of our novel method to study a chromatin of a single locus in single cells. We show further proof of how Tat-dependent transcription elongation is crucial for proliferation of the HIV-1 reservoir.

2. Point-by-point description of the revisions

This section is mandatory. Please insert a point-by-point reply describing the revisions that were already carried out and included in the transferred manuscript.

Reviewer #1 (Evidence, reproducibility and clarity (Required)):

In this manuscript Lindqvist B et al describe a single cell method, based on Proximity Ligation Asssay (PLA) to visualize the early events during HIV-1 reactivation at the level of the viral genome. For this purpose they use a specific protein Zinc Finger Protein 3, constructed to bind specifically to the viral LTR promoter, expressed in a subclone of Jurkat 5A8 cells (with latent HIV-1 full length mutated provirus that encodes for GPF in the frame of Nef) termed 1C10.
The biggest problem which I have here is the specificity of the PLA signal, to which I refer more specifically in correspondence to the specific points listed below. In general terms, although PMA induced cells should have both PLA and GFP signals, there seem to be many cells which have PLA signals only. Moreover, the authors detect a lot of signal in the cytosol in some of the induced cells (at longer time points) to then decide to look only at time points up to 16 hrs.
Despite the fact that figures 1-3 are dedicated to the new method , I do not feel that this methodology represent a well enough controllable system to be used as a novel single cell methodology that allows mapping the chromatin events at the viral promoter. The authors need to explain and comment these setbacks of the methodology.
All of these points are specifically addressed below.
Nevertheless, there are interesting aspects in the manuscript, and the authors may consider further comments below useful for revision:

Related to the Introduction:

1. Line 58: "A well-studied reservoir fraction contains proviruses integrated in active regions, where the HIV-1 promoter is associated with the H3K4me3 chromatin mark."
2. Line 58: It is unclear to which chromatin the Authors refer to. First it is stated that it is an HIV-1 promoter that is marked by H3K4me3, and then that it is the chromatin of the host. Both references suggest open chromatin of the host, but Bativelli does not investigate H3K4me3 at all. Chen et al, on the other hand refer to host chromatin only, where H3K4me3 is further away from the chromatin (103-105 bp). Could the Authors explain this discrepancy?
We have now rewritten and expanded this section to better describe the different chromatin compartments where HIV-1 is integrated, including more references.
3. Line 60- update with the most recent reference Zila et al Cell 2021 This reference has been updated.

4. The sentence 'Even intron-containing HIV-1 transcripts may be exported through the nuclear pore once chaperoned by the viral Rev protein (Coyle et al., 2011)' is placed somewhat out of the context for the presented narrative.
This sentence has been removed.
5. Line 65. 'Stochastic induction of latency is reversed upon T cell activation' Here it is unclear how induction could be reversed. Could the Authors separate these two concepts, the stochasticity of latency establishment and the latency reversal?
Thank you for pointing out this erroneous statement, we meant to say that "the reversal of latency is induced". This is now corrected.
6. 65-67. Reversal from latency is very simplified and seems for a broader audience, popular science.
We have removed these introductory sentences and now present a more focused introduction to latency reversal.
7. 67-onwards should be separated in a different paragraph as it deals with chromatin marks. What is 'gene-like structure' referring to?
We have separated the paragraphs as suggested and provide definitions of "gene-like" and "enhancer-like" chromatin structures.
8. 72-73. encapsulated should be replaced with embedded This replacement is done.
9. 78-79. Are the Authors referring to the clonal expansion of the cells? What does take on mean? We have rephrased the text.
10. 83-84 RNA sequence should be replaced with Trans-activation response element (TAR) We have added the 'trans-activation response element (TAR)' to the text.

Figure 1:

11. Figure 1B: The western blot image is not the best representative image. Both anti-FLAG and beta actin signals in 1C10 cells -/+ PMA/i are unsatisfying. In addition, the actin signal is bleached. We have performed new western blots and replaced the previous images.
12. It would be easier for the reader if different colors (even without patterns) for the bars would be used (as in fig 1F and G). The patterns are difficult to distinguish. We have added colors to the bar charts as suggested.
13. Enrichment in 1C is still lower than expected based on the specificity, at least compared to gag pull

down This is the enrichment we observe in repeated experiments. This ChIP experiment is technically difficult as the FLAG-antibody is expected to only pull down a single short region, resulting in low yields of DNA to purify and then amplify with PCR. However, the binding specificity of ZFP3 to this DNA sequence is already published (Wang et al, 2014). With the ChIP-seq experiment we show that ZFP3 does not consistently bind strongly to any other genomic region. This is novel and has not previously been published.

14. ChIP criteria after peak calling for peak selection are too stringent. We now use the same, more lenient criteria for both ChIP-seq experiments, with a peak selection of FDR threshold at 10^{-5}.

15. 143-146 should be only in the methods part. These sentences are now moved to the Methods section.

16. 150 also should be moved to methods 'staining and mounting'. We have moved the text as suggested.

17. What are the control experiments the authors used to set the arbitrary threshold for PLA intensity?
The threshold was set to find the optimal difference between Tat-ZFP3 PLA spots in DMSO and PMA/i activated cells, while also taking into account the signal in ZFP3 negative cells (5A8). We have reworded the text and Fig S1B has been replaced.

18. How do the authors explain that in some of the PMA induced cells they detect the orange (red) dots of the PLA signal but do not detect the GFP signal in the same cell? This is partially addressed in line 147/148 where the authors claim that the PLA protocol obscure the GFP signal but then they use the antibody to overcome this. Although it might well be that the permeability can vary among cells and therefore the PLA can be detected in some cells where the GFP signal is still obscured (even after an antibody), the authors also hint to a different scenario which is mentioned in lines 223-224. There they suggest a possibility according to which the GFP signal is only partially mediated by Tat-dependent transcription (and PMA induction perhaps in this context). Clarifying this issue is extremely important especially because the authors then in Figure 3D describe a fraction of nuclei with multiple PLA signals in the cytosol (rows 240-251), which further raises the question of the specificity of the method.
This is a valid point and we have now elaborated on this topic in the discussion. The observed frequency of GFP+ cells after PMA/i stimulation correlates well with results from flow cytometry that we have generated previously as well as published results for the 5A8 J-lat cells. The GFP+ fraction after PLA is somewhat higher than from flow cytometry, but in contrast to the flow results, using microscopy we do not exlude dead cells by live/dead stain. From this we conclude that we capture most of the GFP+ cells. For the Tat-ZFP PLA+ cells, we set an arbitrary but conservative threshold based on the results from negative controls from 5A8 cells (that do not express FLAG) and unactivated 1C10 cells (that do not express Tat). As a consequence of using a conservative threshold, we do miss some cells that should have been PLA+, i.e. as the referee point out, GFP+ cells with Tat-dependent transcription. Arguments against this being a major concern are: 1) Making the threshold for PLA more lenient does not include cells with higher GFP levels (Fig S1D); 2) The kinetics of Tat-ZFP3 PLA+ cells are different from the GFP+ cells (Fig 3C–E); 3) There are significant differences in the cell cycle stage of GFP+ and Tat-ZFP3 PLA+ cells (Fig 4E), arguing that the signals represent different entities. However, this can be interpreted as GFP+ cells expressing HIV-1 independent of Tat, or that GFP+ occurs at a different (later) time point than Tat-dependent transcription after proviral latency reversal. Here, we are not able to separate these events as we have not followed live cells in this study. Studying proximity between two single molecules in live cells is technically challenging with today's techniques.
Future development of the technique may lead us to reliably detect ZFP3-Tat PLA spots in more cells. These reflections are now included in the manuscript, but they do not affect the conclusions.

19. 170-171 Figure 1H representation of the data is not appropriate, and should be represented differently or moved to the table. We have removed the Venn diagram and instead present the data as bar charts (Fig 1H). We have also included this analysis for the time series experiments (Fig 3E).
20. Additional time points (later than 16h) should be tested to follow reactivation dynamics In figures 3C–G, 4D–E and S5, we follow the reactivation dynamics during 24 h after T-cell stimulation. We have expanded on the analysis of these late time points in the revised manuscript. As we are primarily interested in Tat-dependent transcription here, late time-points are uninformative.
21. Figure 1I. Pie chart is not the graph of choice to represent the distance, cumulative distance should be plotted at least as a ratio between the dot distance and cell radius, and distance units are meters (m) and not molarity (M). In the revised manuscript we show the distance calculated as distance to nuclear periphery/cell radius as a bar graph instead as suggested. Thank you for pointing out our mistake in units.

Figure 2:
22. In figure 2A cellular lysates were blotted using the anti-Tat antibody, and reference was made to a "well - described" unspecific band. Instead of cropping the blot, the authors should demonstrate the entire region and indicate the specific and unspecific bands. I would suggest also using an additional control protein. We repeated the Tat Western blot but unfortunately we did not manage to get results of better quality, as unspecific bands obscured our blot. Therefore, we have removed this panel and instead provide a reference for this already observed phenotype in J-lat cells.

23. 184 Move the material details of mapping control to the Methods section. This has been moved to the Methods section now.

24. 189. Why is different FDR filtering for peak significance used with respect to analysis of system specificity (Figure 1) We now use the same criteria (see above).

25. Unclear how the six regions were identified, and whether GO statistical overrepresentation was analyzed on those ones only? We are uncertain what the referee is referring to. 1) The six regions of Tat enrichment were identified by MACS2 peaks with an $FDR<10^{-5}$ in both replicates. These regions were within HIV-1 or in intergenic regions, so no GO analysis was possible.
2) We have now clarified in the text that there are the >6,000 regions from the previous publication. GO analysis was done in that reference (Reeder et al 2015) and we now shortly describe what they found.
In both sections, the text has been clarified.

Figure 3
26. In figure 3C the ticks on the y-axis are missing. We have added tick marks.
In rows 240-251 the authors describe multiple PLA signals, some of which observed in the cytosol. Although by using the RNA Immunoprecipitation approach they show that the pulled down Flag-ZFP3 associates with the RNA from the LTR region (and hence prove that the PLA recognizes cytosolic events) they decide to

overcome this problem by looking at earlier time points before the cytosolic RNA starts to accumulate (16hrs). I find this part really hard to understand in the context of the PLA methodology, which they propose as a novel single cell method. Namely, number of Latency Reactivating Agents (LRAs) would require longer exposure times, and perhaps also several rounds of treatment. Therefore having a method that can only be used at shorter time points (up to 16 hrs) is not really an advantage here. The authors should comment on this, as this is a major point of the manuscript.

We do not regard this as a technical problem, but as a biological finding. The Tat PLA method identifies proviruses where Tat in found close to the HIV-1 promoter, which is crucial for physiological activation and viral production. As the reviewer points out, the cytoplasmic dots reduce the use of Tat PLA to achieve this at later time points, when Tat is expressed even after initially Tat-independent transcription. This is a consequence of the biology, and it enriches our understanding of HIV-1 latency reversal and the mechanisms controlling it.

Admittedly, PLA using Tat-ZFP3 indeed has a background (1.5%) higher than we had hoped for. This is a drawback with the method that we now state clearly in the new Discussion paragraph outlining Limitations with the study (lines 465-490). We believe this background signal is a consequence of non-specific binding of the anti-Tat antibody. Anti-Tat antibodies are notoriously poor at recognizing their assigned target, probably because of the relatively large unstructured region of Tat. Using other antibodies, such as HP1α or HP1γ resulted in a 10-fold lower background. In this manuscript, we present an optimized PLA protocol that significantly reduces background signal. It is also a concern that, due to the background, we set an arbitrary threshold for detection of the PLA signal. If we can reduce the background further in future experiments, we might need to adjust the number of cells with Tat at the HIV-1 promoter. This will affect the absolute numbers but the conclusions regarding the kinetics and the mechanistic details described here will not be affected.

Lastly, an important point of the paper is that we detect Tat signal in nuclei of GFP negative cells. The cytoplasmic spots are not background but arise as a consequence of the ZFP3 specifically binding HIV-1 RNA, which also associates with Tat. To confirm the RIP results showing that the ZFP3 protein specifically bind the HIV RNA (Fig 3G) we have now also performed RNA EMSA (electrophoretic mobility shift assay) (Fig S4, lines 270-281). The cytoplasmic foci specifically appear at late time points, and not in unactivated or early after PMA/i activation. This indicate that the PLA signal indeed is specific, and points to a biological effect.

Figure 4

27. Nuclear size as a proxy for cell cycle stage is not an optimal cell cycle phase identifier, and other methods such as DNA content should be explored

We have now recalculated these measurements and use DNA content as suggested (Roukos et al 2015). We attempted to use the Watson model to deconvolute the different phases of the cell cycle, but opted to keep the use of more simplistic thresholds to be able to discern GFP+ and PLA+ cells.

Figure 5.

28. GNE049 control fot Ac on histones by western blotting should be included.
We have included Fig 6A with a western blot of H3K27ac (and H3 as control) after treatment with two doses of GNE049 as well as JQ1.
29. GNE049 might also affect Tat acetylation and this should be commented
We have commented on the possible effect of GNE049 and JQ1 on Tat acetylation in the discussion (lines 403-406).
30. What is the concentration of GNE049 used in the study? Depending on the concentration, the drug could have an effect on BRD4, not only on P300.
Thank you for pointing this out! In the initial manuscript we used a high concentration (10 μM) that indeed also affects BRD4. We have now included a lower CBP/P300-specific concentration of GNE049 (0.5 μM) and compare it to JQ1 that specifically inhibits BRD4. The Tat-ZFP3 PLA results from the low dose of GNE049 showed no significant effect. The results show that Tat-dependent transcription is also blocked by JQ1. We further elaborate in this in the discussion of the revised manuscript. BRD4 has been implicated in HIV latency reversal in many studies.
31. Lanes 339 to 346: The conclusions the authors draw here on tat binding to structures during splicing are incomprehensible based on experiments the paper provide
We agree and have removed this section.
32. In discussion, lane 364: trp should be TPR
This has been removed, in line with the previous comment.
33. Lines 362-364: "Elimination of $HP1_\gamma$ has previously been linked
to HIV-1 latency reversal (du Chene et al., 2007). The PMA/i-induced GFP in the cells with H3K9me3 at the provirus (Fig 5C) might be explained by the presence of $HP1_\gamma$'. Would the presence of $HP1_\gamma$ then not imply that latency is preserved and no GFP is expressed?
In response to this comment we conducted PLA with ZFP3 and HP1, using both the HP1α and HP1γ isoform. In these experiments, we could only detect very low, if any HP1 protein. We have therefore removed this section in the discussion.
34. Line 388 'We show here that, by using small molecules to eliminate the enhancer functionality of the provirus, Tat-independent HIV-1 latency reversal potentially eliminates this reservoir fraction' This conclusion was already reached in the previous study from the Authors (Lindquist et al 2020), Figure 7 and S7.
In our previous papers (Battivelli et al 2018 and Lindqvist et al 2020), we observed that H3K27ac/H3K4me1 was associated with proviral latency and reactivation. In this study, we describe how this reservoir fraction is maintained and possible ways to eliminate it. The requirement of H3K27ac and BRD4 for Tat-mediated transcription of activation of enhancer-embedded proviruses was possible to detect through our novel PLA-based method.

Reviewer #1 (Significance (Required)):

I am not sure that this work will be of interest to a broad audience, because of the methodology that is described here. Although the scope was to develop a method to allow for single cell monitoring of chromatin events at the level of the integrated viral promoter upon reactivation, I have several concerns regarding the

method that need to be addressed by the authors.
The method is supposed to represent the innovative was to monitor viral latency (and the reactivation of it) at the single cell level; otherwise the rest of the data albeit potentially interesting are not sufficiently developed.
We appreciate your comments and in the revised manuscript we have developed the data substantially. To make it of interest to a broader audience, we have also included a more general model where any genomic region is marked by a dCas9 protein that can be queried for chromatin environment in single cells (lines 190-197, Fig S2A–B). Also, this methodology can be adapted to other systems, i.e. one can design a ZFP (or simply dCas9) that binds to any genomic location and study the chromatin microenvironment, e.g. *c-myc* in cancer cells.

Referee Cross-commenting

The single cell technique that the authors developed to evaluate the mechanisms of HIV latency reversal in a cell line model is based on the proximity ligation assays (PLA), which allows them to detect the presence of selected proteins, in particular the viral transactivator Tat protein at the viral LTR promoter. While I agree with Rev 2 that the method is innovative, I don't necessarily think that it is widely applicable, and most of all I have serious concerns about the specificity (and high background). I also found it disappointing that the authors did not recognize these points and did not discuss them in more details. I also tend to think that the study might be more appropriate for a specialized journal, however it will be up to editors to decide.

In the revised manuscript we have addressed the issue of background and specificity. Despite these short-comings, there is no other current generally available method to study chromatin and single molecules in single cells. The conclusions from this study regarding mechanistic insight into HIV-1 latency reversal are not affect by the background and specificity issues, and these results could not have been reached using other currently available methods.

Reviewer #2 (Evidence, reproducibility and clarity (Required)):

The authors describe an elegant single cell technique to evaluate mechanism of HIV latency reversal in a cell line model. They transduce a previously described zinc-finger protein (ZFP) that binds specifically to the HIV LTR into transformed T cells carrying an integrated HIV genome encoding a GFP reporter. They are then able to perform proximity ligation assays (PLA) to detect the presence of selected proteins at the LTR, focusing on the viral transactivator Tat. In cells stimulated with PMA+ionomycin (PMA/I), they detect Tat at the HIV promoter in a small fraction of the cells, only some of which are expressing viral proteins (as measured using GFP). A larger fraction of cells are GFP+, PLA-. The authors use this system to make a variety of observations about ZFP binding to RNA, cell cycle arrest, activity of latency reversing agents (LRAs), and heterochromatin marks at the LTR, none of which are fully pursued.
Overall, this is a very interesting and potentially important manuscript. The authors are to be congratulated for developing an elegant system for detecting proteins at the HIV LTR. The manuscript could be improved by considering the following points:

1) It is important to acknowledge that the experiments were performed in a continuously proliferating cell line with an HIV reporter virus integrated into a single position in the human genome. The authors could discuss in more detail how findings in the model relate to the in vivo situation where latent HIV resides in resting CD4+ T cells that are in G0 with different integration sites in each infected cell or infected cell clone. Throughout the manuscript, the authors should make an effort to cite and discuss papers in which similar issues have been explored using cells from infected individuals. For example, the differential inducibility of different proviruses, the poor ex vivo activity of many LRAs, etc.
We agree. Additional references are included and we in the revised we bring up all the issues the reviewer mentions.

2) The Tat immunoblot in Figure 2A could be improved.
We have removed this immunoblot as we were unable to improve the bands. Instead we provide a reference in the text for this established result.

3) The authors could provide a more detailed explanation of the observed Tat-independent GFP expression. For example, could it be due to low sensitivity of the PLA method for detecting Tat as the LTR? Alternatively, could a larger portion of HIV gene expression be truly independent of Tat?
We have now expanded on this issue and included these points in the revised paper, both as new analysis and in the text.
The authors could also try to relate this observation to in vivo data showing that most proviruses are defective, often with large deletions affecting the 3' end of the genome, including that tat gene. There is increasing evidence for viral gene expression from defective proviruses.
Also these points have been incorporated in a new discussion section (lines 470-476). The large fraction of defective proviruses in the reservoir of PLWH make it even more important to have reliable models to identify and study mechanisms of latency reversal of intact proviruses that are able to reseed the infection.

4) The suggestion that the ZFP bind to RNA should be validated more fully at the biochemical level.
We have now performed RNA EMSA in addition to the RNA-IP previously used (Fig S4, lines 270-281). These results confirm that, in addition to the HIV-1 LTR DNA sequence, ZFP3 also binds to the RNA sequence of the HIV-1 LTR.

5) The analysis of different latency reversing agents in this system is interesting and clinically important and should not be relegated to the supplementary material.
Point taken and we have moved parts of the LRA results into a main figure (Fig 3A–B).

6) The authors hypothesize that activation-induced cell death could account for some of the observed effects, but this idea is not pursued in any detail. PMA/I is often very toxic to cells and it seems important to understand the fraction of cells that die, whether the death is due to viral gene expression, and whether other modes of T cell activation produced similar effects.
We have further explored the cell cycle arrest and cell death experimentally using EdU-pulsing and Annexin V/PI staining. We have also refined the previous analysis of the PLA results. By comparing the HIV-1

containing Jurkat clone 5A8 with the parental Jurkat cells, we can attribute excessive cell death to apoptosis, and specifically distinguish the effects of the viral toxicity and T cell activation. We are able to attribute a G2/M arrest to HIV-1, whereas the G1/S block is largely due to the PMA/i-mediated activation as it occurs regardless of HIV-1. Other activating agents, anti-CD3/CD28 or prostratin, did not affect cell death. Further, prostratin–now included as an LRA–did not result in significant Tat-dependent HIV-activation.

Reviewer #2 (Significance (Required)):

Please see above.

Referee Cross-commenting

I believe that the technique developed here may be important for the field and that the paper could be acceptable if most of the reviewer comments are addressed.
Thank you for your insightful comments that have all been addressed to improve the manuscript.

Reviewer #3 (Evidence, reproducibility and clarity (Required)):

T cell stimulation remodels the latently HIV-1 infected cell population by differential activation of proviral chromatin
Birgitta Lindqvist
Review Commons

On ART proviral transcription is observed without leading to transcripts translated in an immune escape manner. CTL from EC however can kill cells exposing viral proteins in combination with most proviruses integrated in repressive chromatin. Therefore, CTL function tailor HIV reservoir size and function is inversely correlated with inflammation or T Cell exhaustion or macrophage activation.
Strategy to induces latency reversal were disappointing as due to several positive feedback loops, including Tat, that can self-regulating its own transcript while HIV-1 can induce cell death in absence of cell infection and therefore cell clearance in turn activates HIV-1. Using GFP as a marker of total viral activation one can distinguish productive, Tat-dependent HIV-1 activation from in vitro induced Tat-independent activation. Investigators assessed in a single cell method chromatin at the HIV-1 locus and link it to early stage of HIV-1 activation. They showed that during T cell stimulation, Tat was transiently recruited to the HIV-1 promoter only when cell proliferate. Following T cell stimulation, the proviral chromatin landscape within the HIV-1 infected cells led to cell death. Importantly, Tat was present only at the HIV-1 promoter when it had enhancer features delivered by CBP/P300. By using small molecules to eliminate the enhancer functionality of the provirus, Tat-independent HIV-1 latency reversal may represent a new approach to reduce reservoir size.
The proposed model is interesting from an in vitro perspective. However how the proviral chromatin composition will affect the latently HIV-1 infected T cells after stimulation and non-infected cells after several stimulation.
What can be the off-target effect of CBP-P300.

The off-target effect of GNE049 as an inhibitor of CBP/P300 is that it also inhibits BRD4. BRD4 is acting downstream of CBP/P300 and we have now included BRD4 inhibition in the manuscript. Using the BET inhibitor JQ1 we observe a similar but less pronounced phenotype regarding Tat recruitment.
The CBP/P300 in itself has numerous non-HIV targets. CBP/P300 act as a general acetyltransferase in the genome and inhibition would not be specific for the HIV provirus. CBP/P300 inhibitors have not been tested in clinical trials yet to our knowledge. BET inhibitors, including analogues of JQ1 to target BRD4, have been tested clinically. These agents are well-tolerated in patients and some have showed success in treatment of hematological maligancies including acute myeloid leukemia (for a recent review, see Shorstova et al, BJC 2021 124:1478). This would make it possible to repurpose anti-cancer drugs toward anti-HIV drugs, for a rapid translation of our finding to the clinic.

Effect on subset memory T cells will help to understand what the T cell activation can induced with cells having baseline different level of activation and chromatin structure.
We have further analyzed the different levels of activation by comparing PMA/i-mediated T cell stimulation with stimulation through CD3/CD28 antibodies and prostratin. We further discuss more in depth the different chromatin structures and in Fig 6 we have included different doses of the GNE049 drug and also BET inhibitor JQ1 that act on the downstream target BRD4.

Following T cell stimulation, the proviral chromatin landscape within the HIV-1 infected cells was altered due to selective cell death. Types of cell death are very numerous and different pathways are involved. To further illustrate the mechanism of cell death related to the T cell stimulation.
In the revised manuscript we show have quantitated cell death using Annexin V/PI staining and flow cytometry (Fig 4A, Fig S5B). The results show the cell death is induced through apoptosis, and that apoptosis occur as a consequence of PMA/i mediated cell activation and also cytotoxic effects of HIV-1. The effect on Tat was now quantitated with a new analysis of the subG1 peak (representing dead cells) in the time course (Fig S5D). To further dive into these questions, we also performed dedicated cell cycle experiments and detected a general cell cycle arrest in G1 and a HIV-1 specific arrest in G2.

Reviewer #3 (Significance (Required)):

Very difficult to inferred from this in vitro model to animal model effect like in humanized mice
We acknowledge the need of follow-up studies in other *in vivo* models. We have further connected our data to several other studies performed in PLWH. Also, the significance we provide mechanistic insight to a potentially important mode of HIV-1 reactivation. We also present evidence as to why the latency agents that have been used so far have not been successful in clinical trials (i.e. the lack of Tat-mediated activation). Additional significance come from the presentation of a unique technique that allows single cell characterization of chromatin at specific chromatin loci.

Referee Cross-commenting

As per reviewer 1 and 2 and myself the relevance of study findings in cells been in G0 and different Cd4

memory subtypes are nor addressed in the cellular model, that may limit interest for humanized mouse models.
Other models and follow-up studies from other labs will be required to validate our findings. However, important to keep in mind, whereas the reservoir persists in resting G0 cells, the reservoir is maintained by proliferation of HIV-1 infected cells. Even though the reservoir cells are not actively dividing, HIV-1 latency reversal is favoured in metabolically active cells. During the steps of reseeding or clonally expanding the reservoir, CD4 cells will acquire features resembling cycling J-lat cells. The results from our study provide a link to the mechanisms underlying observed reservoir fluctuations and proviral chromatin in PLWH (Jiang et al Nature, 2020 585:261, Einkauf et al Cell 2022 online Jan 12).

May better fit a molecular journal of virology, as your readership might be less specialized
We have now included other aspects, such as a generalization of the method using dCas9 that can be directed to any genomic locus. As there currently are few options in characterizing chromatin in single loci in single cells, this widens the target audience substantially to include non-HIV scientists.
Thank you for the comments. We believe that they have substantially improved the manuscript and made it more accessible for a general audience.
