# Supplementary material for: T cell stimulation remodels the latently HIV-1 infected cell population by differential activation of proviral chromatin
Source: PLoS Pathog. 2022 Jun 6;18(6):e1010555. doi: 10.1371/journal.ppat.1010555 (PMC9203004; doi:10.1371/journal.ppat.1010555)
Supplement: Attachment — Submitted filename: rebuttal2_3.docx [file ppat.1010555.s009.docx]

We thank the reviewers again. In addition to adding the changes requested by reviewer 2, we have also included a Summary paragraph in the new manuscript.

Reviewer #2: The question about the specificity of the method has already been raised previously, and there (point 18 reviewer 1) the authors argued that the GFP expression occurs at different (later) time point with respect to Tat binding. This is indeed plausible, as shown also in Figure 3E, lines 257-259. However, while the levels of GFP continue to increase, Tat-ZFP3 PLA signals peak at 18hrs and then show a sharp drop. Therefore the authors opt for 16 hrs but the discrepancies remain, with their explanation being that the discrepancy between Tat-ZFP3 and GFP can be due to two mechanisms:

-transient association of Tat with the TAR early in latency reversal followed by GFP accumulation
-Tat-independent mechanisms leading to GFP expression

This is a problem, because this leaves the authors with no appropriate controls for the PLA assay.
The best way to control Tat induced transcriptional activity of the LTR promoter is to actually measure HIV-1 transcription by RT-PCR using different primers, for short (abortive) transcripts as well as long ones.

The other way to control their experiments if to measure the number of viral particles, as the 1C10 cell produce and bud off non-infectious viral particles after activations (lines 266-267).

We control the PLA assay in different ways. Qualitatively we use parental 5A8 cells as negative control for Tat-FLAG PLA, and 1C10 cells after PMA/i stimulation as positive control. Quantitatively, we control the assay using PLA with antibodies against FLAG and H3. Theoretically, FLAG-H3 should yield a signal in all cells, but the sensitivity of the assay lets us only detect a fraction of the cells as positive. We have opted for an arbitrary but conservative threshold as not to score background, and optimization of the protocol may necessitate us to revisit the actual numbers reported.

Tat-induced transcription of the LTR promoter is well described by us and others in the literature. We have previously measured the HIV-1 transcription in these cells (the parental J-lat 5A8) using RT-PCR. In Fig 1E (with/without PMA/I activation) and Fig 7F (after pretreatment with GNE049) of our previous paper Lindqvist et al, 2020 (Plos Pathogens) the requested controls are available. The GFP levels in J-lat cells correspond extremely well to HIV-1 RNA in single cells, as we have shown using RNAscope (Zhang et al 2018). We now refer more clearly to these data in the revised manuscript (lines 144-146).

**Fig 1: Viral RNA cannot be reliable detected in the supernatant.** Cells were seeded in normal media and pretreated with DMSO or GNE049 (10 uM) for 3 h, before addition of DMSO or PMA/i. After 24 h supernatant was harvested and viral RNA isolated (Qiaamp Viral RNA mini Kit). cDNA was constructed using Revertaid reverse transcriptase (ThermoFisher). q-RT-PCR was performed with Powerup Sybr green (ThermoFisher on an Applied Biosystems 7500 Fast Real-Time PCR system. Primers against 5´LTR (Table S2) were used. Data was normalized to genomic DNA by ∆Ct. Four independent experiments were performed and error bars represent s.e.m..

In the previous manuscript version, we stated that the 1C10 cells bud-off non-infectious viruses. In response to the reviewer’s comments, we sought to quantitate viral proteins and RNA in the supernatant. In these new experiments suggested by the reviewer, viral protein p24 was under our detection level even after 24 h PMA/i stimulation. For viral RNA (Fig 1 of this document), the HIV-1 genome detected in the supernatant by q-RT-PCR was inconsistent. Unfortunately, this means that we are not able to use this method in the J-lat cells to draw conclusions on virus budding. As a consequence, we removed the statement of 1C10 cells shedding viruses (line 278). We thank the reviewer for making us aware of this and thereby strengthening the study.

These new data do not diminish our findings though as almost exclusively the Tat-PLA spots were observed in the cytoplasm of cells after 24 h PMA/i stimulation.

Other minor points are listed bellow:

• The discrepancy between the microscopy and flow cytometry data (rows 175-177 of the Msc,) could be interpreted as sensitivity of the microscope based detection, but unlikely are due to compromised cellular viability, simply because dead cells will not adhere to the slides. As these are Jurkat cells, which need to be adhered to slides, dead cells will simply not attach to slides.

Thank you for pointing this out. Indeed, dead cells do not adhere to the slides. We have removed the last part of the sentence in the ms “The discrepancy between GFP+ rates from microscopic imaging and flow cytometry indicates that the microscopy settings are more inclusive and detect cells with lowly expressed GFP ~~and possibly cells with compromised cell membrane due to early toxic effects of the viral proteins or the treatment~~” (line 189).

• It is clear already from data in Figure 1 (Figure 1E) and then later from Figure 3A and 3B that there are significant differences between Tat-ZFP3 PLA results and expression of GFP in the same cells. From the results presented in Figure 3, where different LRAs were used, it seems that GPF detection scores higher for the detection capacity of HIV-1 activation. Number of GFP + cells in PMA/i is almost 30% whereas there are less than 6% of PLATatZFP3+ cells. Also, when LRA activity was compared, it seems that the all the LRAs give some background PLA signal, while the number of real positive cells is low. Differences between LRAs are much better appreciated with GPF as a readout. How were these signals normalized? The authors need to add HIV-1 mRNA levels as a readout. They can also specifically measure the GPF signal as mRNA level

The data is not normalized but reported as detected (“% cells with a single PLA+ focus”). We agree that the PLA method, given its narrow dynamic range, is not well suited for large-scale comparisons of LRA activity. It is however well-suited to a targeted delineation of mechanisms behind HIV-1 reversal, such as our study of GNE049 here. As we clearly stated, the background of the PLA is higher than the GFP. As we previously have shown (Zhang et al 2018, referred to above and in the revised manuscript), the GFP levels is a suitable proxy of mRNA levels, arguing against repeating the entire experiment to isolate and quantitate mRNA.

• Chromatin signatures of the PLA positive cells remain very puzzling. Considering the fact that in PLA + cells GFP intensity increase upon PMAi induction concurrently with all histone marks , both activating ie K27ac and K4me3, as well as repressing, K27me3 and K9me3 is further complicating the interpretation of these data.

We agree that the biology behind these data is puzzling. The data show that even repressive marks do not fully protect against HIV-1 latency reversal in these cells. Of note here, we did not detect HP1 at the H3K9me3 embedded proviruses (lines 353-357). Interconnected HP1 molecules act as a shield to the chromatin and, without HP1 the chromatin remains exposed at the provirus in these cells. H3K27me3 is a mark of facultative heterochromatin and a strong inducer such as PMA/i is able to override these repressive signals.
